# Duodenal Adenocarcinoma Versus Foreign Body Granuloma in the Background of Postcholecystectomy Migration of Endoclip Into Duodenum: A Histopathological Surprise

**DOI:** 10.7759/cureus.23086

**Published:** 2022-03-11

**Authors:** Arkadeep Dhali, Sreecheta Mukherjee, Arunesh Gupta, Sukanta Ray, Gopal Krishna Dhali

**Affiliations:** 1 Gastrointestinal Surgery, School of Digestive and Liver Diseases, Institute of Postgraduate Medical Education and Research, Kolkata, IND; 2 Gastroenterology, School of Digestive and Liver Diseases, Institute of Postgraduate Medical Education and Research, Kolkata, IND

**Keywords:** foreign body granuloma, duodenal adenocarcinoma, cholecystectomy, migration, endoclip

## Abstract

A surgical endoclip in the cystic pedicle rarely migrates to the duodenum and is considered a rare complication of laparoscopic cholecystectomy. Duodenal adenocarcinoma endoscopically mimicking a foreign body granuloma in the background of postcholecystectomy endoclip migration has never been reported before.

A 53-year-old Indian male presented with progressive weakness and melena for the last three months. He underwent laparoscopic cholecystectomy a year ago with an uneventful clinical course and post-operative recovery. A complete hemogram revealed hemoglobin of 4.5g/dL. Upper gastrointestinal endoscopy revealed a large necrotic polypoidal mass arising from the lateral wall of the first part of the duodenum. Contrast-enhanced computed tomography (CT) of the abdomen showed an impacted surgical clip into the lateral wall of the first part of the duodenum. Intraluminal extension of the surgical clip was not appreciated in the imaging. We suspected the diagnosis to be foreign body granuloma in the duodenal wall. He underwent open duodenal wedge resection. Microscopic evaluation of resected specimens revealed poorly differentiated adenocarcinoma. All the resection margins were free. He had an uneventful recovery and was discharged on the seventh post-op day. He was symptom-free and doing well on follow-up at 12 months.

The purpose of reporting the case was to make the readers aware of the delayed massive upper gastrointestinal hemorrhage as a rare complication of endoclip migration (ECM) post laparoscopic cholecystectomy. In our case, the duodenal adenocarcinoma mimicked a foreign body granuloma endoscopically, and hence a possibility of duodenal adenocarcinoma as a potential delayed complication of ECM cannot be ruled out.

Although rare, in case of upper gastrointestinal hemorrhage in the background of the previous history of laparoscopic cholecystectomy, endoclip migration should be kept as a differential diagnosis.

## Introduction

Laparoscopic cholecystectomy (LC) is performed worldwide for symptomatic cholelithiasis. A surgical endoclip in the cystic pedicle rarely migrates to the duodenum and is considered a rare complication of LC. These endoclips can erode the duodenal wall giving rise to ulcers that cause upper gastrointestinal bleed [1]. Herein, we present a novel case of poorly differentiated duodenal adenocarcinoma mimicking foreign body granuloma manifesting with massive upper gastrointestinal bleed. Existing medical literature is sparse with non-standardized clinical management, and little is known about clinical outcomes. Due to the rarity of such clinical dilemmas, observational studies are not feasible. Hence it is of paramount importance to report such cases. The case report was realized according to international Surgical CAse REport (SCARE) guidelines [2].

## Case presentation

A 53-year-old Indian male presented with a progressive weakness for the last three months, which started with one episode of the passage of dark tarry stool, which was not associated with abdominal pain, jaundice, vomiting, fever, or weight loss. He did not have any history of alcohol abuse. He was diabetic and hypertensive for the last five years and took medication for the same. He underwent laparoscopic cholecystectomy for symptomatic gallstone disease a year ago with an uneventful clinical course and post-operative recovery. Further details of the procedure were not available.

**Figure 1:**
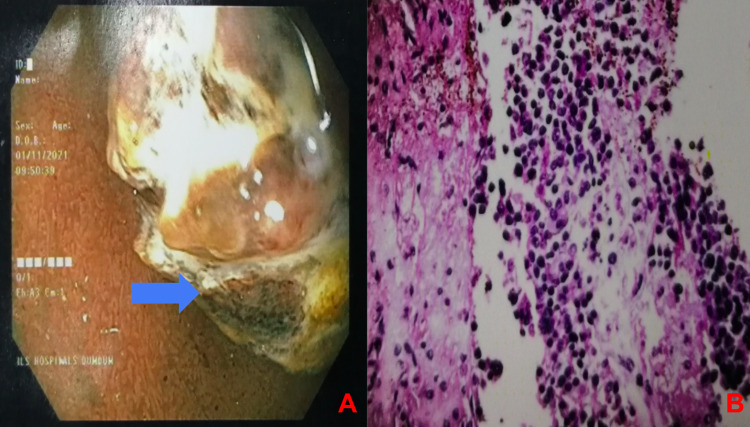
(A) Upper gastrointestinal endoscopy showing a large necrotic polypoidal mass (blue arrow), arising from the lateral wall of the first part of the duodenum, with no evidence of ulcer or active bleed (B) Endoscopic biopsy showing abundant areas of hemorrhage and necrosis with few clusters of small round cells with no nuclear pleomorphism.

**Figure 2:**
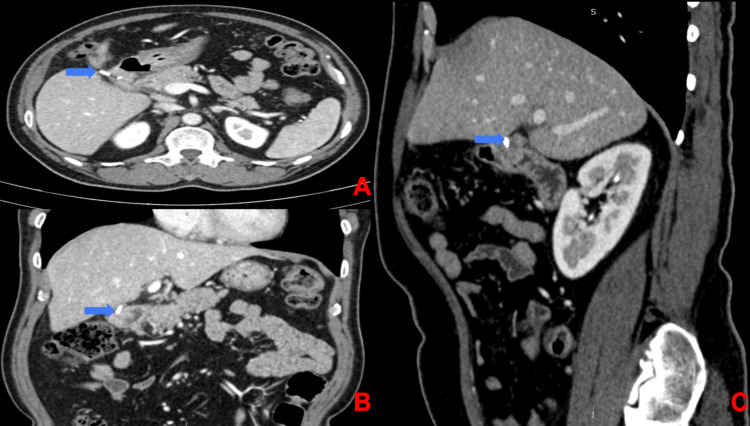
Contrast-enhanced computed tomography (A: transverse section, B: coronal section, C: sagittal section) of the abdomen showed an impacted surgical clip (blue arrow) into the lateral wall of the first part of the duodenum with nodular soft tissue adjacent to it and minimal pyloroduodenal thickening.

**Figure 3:**
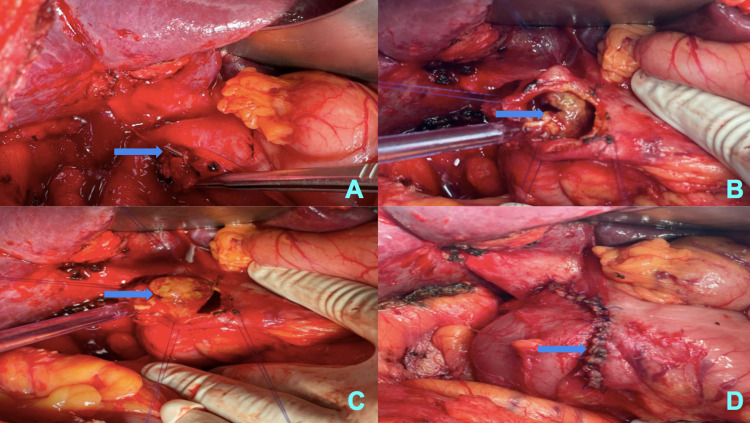
Intraoperative image showing (A) surgical endoclip (blue arrow) impacted on the first part of the duodenum (B, C) globular mass (blue arrow) hanging from the lateral wall of the first part of the duodenum (D) primary repair after wedge resection of the first part of the duodenum (blue arrow).

**Figure 4:**
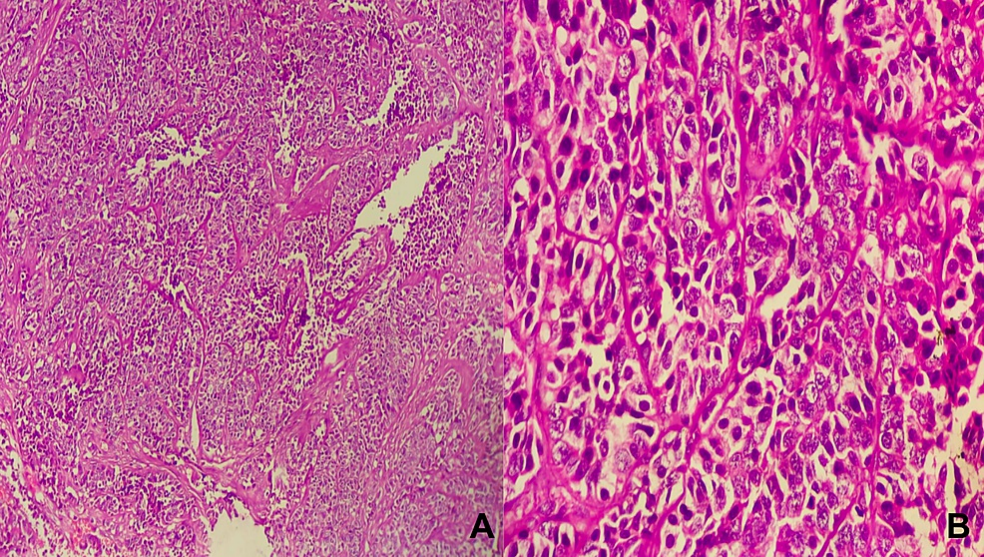
Hematoxylin and eosin images showing (A: 40X, B: 100X) poorly differentiated adenocarcinoma infiltrating duodenum from the serosal aspect.

Physical examination revealed pallor, tachycardia (pulse rate: 110/min), and hypotension (100/60 mmHg). A complete hemogram revealed hemoglobin of 4.5g/dL. Other biochemical investigations such as liver function tests, serum electrolytes, serum amylase, and lipase were within normal limits. Initial resuscitation was done with 4 units of packed cells. Post-transfusion, hemoglobin was 10.2 g/dL. Ultrasonography of the abdomen was unremarkable. Esophagogastroduodenoscopy revealed a large necrotic polypoidal mass arising from the lateral wall of the first part of the duodenum, with no evidence of ulcer or active bleed (Figure 1A). Endoscopic biopsy (Figure 1B) revealed abundant areas of hemorrhage and necrosis with few clusters of small round cells. Nuclear pleomorphism was not seen. Contrast-enhanced computed tomography of the abdomen showed an impacted surgical clip into the lateral wall of the first part of the duodenum with nodular soft tissue adjacent to it and minimal pyloroduodenal thickening (Figure 2). Intraluminal extension of the endoclip couldn't be appreciated in the imaging. Based on the endoscopic findings and radiological imaging, we suspected the diagnosis to be foreign body granuloma in the duodenal wall caused due to the impacted endoclip. We couldn't rule out malignancy without histopathological evaluation of the resected specimen. Given the provisional diagnosis of foreign body granuloma of the duodenum, he underwent open duodenal wedge resection (Figure 3). Operative time was 135 minutes, and intraoperative blood loss was 50ml. The resected specimen was sent for histopathological evaluation, which revealed poorly differentiated adenocarcinoma (Figure 4) infiltrating the duodenum from the serosal aspect. All the resection margins were free. He had an uneventful postoperative recovery and was discharged on the seventh post-op day. He was symptom-free and doing well on follow-up at 12 months. The surveillance computed tomography imaging and endoscopy showed no evidence of recurrence.

## Discussion

Laparoscopic cholecystectomy (LC) remains the gold standard and widely accepted treatment modality for symptomatic gallstone disease. Endoclips are used in LC for controlling the cystic duct and cystic artery and are reasonably safe and effective. On some rare occasions, they may migrate to cause several complications, such as choledocholithiasis, acute pancreatitis, cholangitis, choledochoduodenal fistula, hepatolithiasis, bile leak, biliary stricture, duodenal ulcer, clip embolism, and sepsis [3-5]. This rare phenomenon was first described by Onghena et al. in 1992 [6]. Existing medical literature shows that the incidence of endoclip migration (ECM) varies from 1% to 15% depending on the extent of dissection of cystic duct performed and the number of endoclips used in attaining biliostasis and hemostasis [7]. In most cases, ECM occurs along the tissue planes with no or minor consequences [8]. Overall, ECM leading to the formation of stone occurs at a significantly longer interval compared with no stone formation (median 36 months vs. 5.5 months) [8]. Some factors contributing to the migration of endoclip include (a) ineffective cystic duct closure by clips (b) infective process localized around the clip (c) inadvertent placement of endoclips in the wall of the bile duct during the initial operation (d) biloma formation (e) cystic duct stump closed by more than four clips (f) cholecystectomy in the background of pancreatitis or acute cholecystitis [9]. In the background of acute cholecystitis, excessive pressure by the clip applicator may cause a cheese-wire effect and necrosis of the cystic duct stump leading to ECM. In this setting, suture ligature of the cystic duct is better, but some surgeons are not acquainted with intracorporeal suture placement [10]. Diagnosis in these cases requires a high index of clinical suspicion. Radiological investigations like computed tomography and magnetic resonance cholangiopancreatography play an important role. Endoscopic retrograde cholangiopancreatography remains the gold standard diagnostic modality with sufficient therapeutic benefits. Both wire baskets and balloon catheters can be safely used for clip retrieval. Surgical intervention is reserved where the endoscopic approach is not feasible or has failed, as in our case. 

The purpose of reporting the case was to make the readers aware of duodenal adenocarcinoma endoscopically mimicking a foreign body granuloma in the background of postcholecystectomy endoclip migration. Hence, the possibility of duodenal adenocarcinoma as a potential delayed complication of ECM cannot be ruled out. Further studies are required to establish this.

Moreover, we couldn't possibly be sure whether there was a coexisting duodenal adenocarcinoma at the time of evaluation. There was no way we could have preoperatively diagnosed this sinister pathology, given the clinical context. Having said that, a high index of clinical suspicion should be there to look for malignancy in the gastrointestinal tract when an adult/elderly patient presents with anemia. 

## Conclusions

Although rare, in case of upper gastrointestinal hemorrhage in the background of the previous history of laparoscopic cholecystectomy, endoclip migration should be kept as a differential diagnosis. Moreover, malignancy should be ruled out by histopathological evaluation before labelling it as foreign body granuloma. A sinister pathology like this can be easily missed unless looked for extensively in all cases of anaemia in an adult or elderly patient.
